# Effects of workplace incivility and workload on nurses’ work attitude: The mediating effect of burnout

**DOI:** 10.1111/inr.12974

**Published:** 2024-04-25

**Authors:** Seung Eun Lee, Ja‐Kyung Seo, Maura Macphee

**Affiliations:** ^1^ College of Nursing Yonsei University Seoul South Korea; ^2^ Industrial and Organizational Psychology Yonsei University Seoul South Korea; ^3^ School of Nursing University of British Columbia Vancouver Canada

**Keywords:** health service management, job satisfaction, nursing, nursing shortage, organizational commitment, turnover, violence in the workplace, work environment

## Abstract

**Aim:**

The study's aim was to examine how workplace incivility and workload influence nurses' work attitudes (turnover intention, job satisfaction, and organizational commitment) using the stress–strain–outcome framework.

**Background:**

There is a lack of comprehensive research on the combined effects of workplace incivility and workload on nurses' work attitudes.

**Introduction:**

Two workplace stressors, incivility and workload, were hypothesized to lead to burnout, which in turn influences nurses’ work attitudes.

**Methods:**

A cross‐sectional, descriptive correlational study was conducted. Survey data were collected from 1,255 direct care nurses with a minimum of 6 months’ nursing experiences in 34 general hospitals across Korea. Structural equation modeling was used to test the hypothesized model. This study is reported using the STROBE checklist.

**Results:**

As hypothesized, both workplace incivility and workload increased burnout. Heightened burnout correlated with increased turnover intention, lowered job satisfaction, and reduced organizational commitment. While workplace incivility impacted these outcomes both directly and indirectly via its effect on burnout, workload influenced the outcomes solely through burnout.

**Conclusion:**

The study's findings are based on one, nonrandomized sample of nurses working at South Korean hospitals. Despite such study limitations, the study findings support the adverse impact of two workplace stressors on burnout and nurses’ work attitudes.

**Implications for nursing:**

Evidence‐informed interventions for both workplace stressors include training programs, clear policy guidelines, open communication channels, and supportive work environments.

**Implications for nursing and health policy:**

Zero tolerance and equity, diversity and inclusivity policies to promote workplace civility must be enforced. Workload needs to be patient‐centered, ensuring a “fit” between patient needs and nurse staffing.

## INTRODUCTION

Nurses face common workplace stressors, such as workplace incivility and heavy workloads, which have negative effects on their physical and mental well‐being and work performance. Workplace incivility, characterized by discourteous or rude behavior with disregard for others (Guo et al., 2022), is unfortunately prevalent in healthcare settings (Martin & Zadinsky, 2022). It can lead to a range of adverse consequences, including increased burnout and decreased organizational commitment (Martin & Zadinsky, 2022), reduced job satisfaction (Samad et al., 2020), and increased turnover intention (Kavaklı & Yildirim, 2022). Similarly, heavy workloads, another common work‐related stressor (Keith et al., 2020), are associated with an increased risk of burnout (Diehl et al., 2021), high turnover (Rotenstein et al., 2023), and decreased job satisfaction (Keith et al, 2020) among nurses.

To better understand how workplace stressors lead to nurses’ negative work attitudes, we employed the theoretical stress–strain–outcome (SSO) framework, which explains how workplace stressors affect work attitudes and behaviors via psychological strain (Fox & Spector, 1999). The term “strain” refers to the negative emotional and psychological impacts of stressors, while “outcome” denotes the consequent behavioral or psychological responses (Koeske & Koeske, 1993). To address research gaps in the nursing literature with respect to underlying mechanisms that link stressors to outcomes, we applied the SSO framework to nursing. We posited that two workplace stressors, incivility and workload, lead to strain, as manifested by burnout, which adversely influences nurse work attitudes. In this study, three work attitude outcomes were turnover intention, job satisfaction, and organizational commitment. Research evidence suggests linkages among the constructs being explicitly tested with the SSO framework in this study. However, limited research has explored the simultaneous impact of workplace incivility and workload on nurses' work attitudes, particularly regarding whether this relationship is mediated by burnout, a strain response. Given the global nursing workforce shortage, it is crucial, therefore, to understand how different job stressors can affect nurses, as some stressors may have more detrimental consequences than others (Hoeve et al., 2019).

Existing evidence includes a meta‐analysis that showed that workplace incivility significantly contributes to employees’ turnover intention, surpassing other forms of workplace bullying (Han et al., 2021). Work overload was also significantly linked to nurses’ burnout (Diehl et al., 2021) and healthcare workers’ turnover intention (Rotenstein et al., 2023). Additionally, burnout was associated with turnover intention among medical doctors (Aman‐Ullah et al., 2023). Thus, building upon the SSO framework and previous research findings, this study proposed the following hypotheses:

Hypothesis 1 Workplace incivility and workload increase nurses' turnover intention, and their relationship is mediated by burnout.

Job satisfaction reflects how well the job meets an individual's expectations and needs. Research has shown that workplace incivility (Khan et al., 2021) and heavy workload (Rostami et al., 2021) negatively affect nurses’ job satisfaction. Moreover, there is a noted association between nurse burnout and decreased job satisfaction (Jun et al., 2021). Thus, we propose an additional mediation path as follows:

Hypothesis 2 Workplace incivility and workload decrease nurses' job satisfaction, and this relationship is mediated by burnout.

When employees are satisfied with their jobs, they are more inclined to display organizational commitment (Callado et al., 2023). Organizational commitment refers to the extent to which an individual identifies with and engages in a particular organization, demonstrating a strong belief in its goals and values and a willingness to invest effort in the organization (Meyer et al., 1993). Prior research reported that workplace incivility negatively affects nurses’ organizational commitment (Martin & Zadinsky, 2022), as does work overload (Tayfur Ekmekci et al., 2021). Additionally, there was a negative link between burnout and organizational commitment (Wullur & Werang, 2020). Building upon these findings, we propose the following hypothesis:

Hypothesis 3 Workplace incivility and workload decrease nurses' organizational commitment, and their relationship is mediated by burnout.

### Aim of study

This study aimed to investigate the relative influence of workplace incivility and workload on nurses’ turnover intention, job satisfaction, and organizational commitment through burnout. By doing so, the study endeavors to gain insights into the interplay and comparative strengths of workplace incivility and workload in determining burnout and the subsequent outcome variables, thereby offering valuable antecedents of burnout and practical implications for mitigating its impact on nurses and the organization as a whole.

## METHODS

### Study design, setting, and participants

This study is part of a larger study examining workplace and patient safety in hospital environments. It employed a cross‐sectional, correlational design, analyzing survey data from 1,255 nurses working in 34 general hospitals across Korea. Random sampling was not possible, given the multiple variables associated with the structure and function of the 34 hospitals. Instead, we purposefully selected hospitals (all over 300 beds) according to their locations across seven metropolitan areas and nine provinces in Korea. Eligibility criteria for survey participation required nurses to have a minimum of 6 months of direct care experience. Nurses in managerial positions were excluded from the study. More detailed descriptions of sampling are published elsewhere (Lee et al., 2024).

### Measures

Workplace incivility was assessed using 14 items from the Workplace Incivility Scale, which has shown good psychometric properties (Cortina et al., 2001). Responses were scored on a 4‐point Likert scale ranging from 1 (never) to 4 (most of the time), with higher scores indicating higher levels of workplace incivility. In this study, the Cronbach's alpha was 0.91.

Workload was assessed using the 3‐item Role Overload subscale of the National Survey of the Work and Health of Nurses Scale (Shields & Wilkins, 2006). Responses were scored on a 5‐point Likert scale ranging from 1 (strongly disagree) to 5 (strongly agree), with higher scores indicating higher levels of workload. In this study, the Cronbach's alpha was 0.77.

Burnout was assessed using the 7‐item Copenhagen Burnout Inventory, which has shown good psychometric properties (Kristensen et al., 2005). Responses were scored on a 5‐point Likert scale ranging from 1 (never) to 5 (always), with higher scores indicating higher levels of burnout. In this study, the Cronbach's alpha was 0.89.

Turnover intention was measured using the 3‐item Turnover Intention Scale used by Sjöberg and Sverke (2000) and later validated by Chirumbolo and Hellgren (2003). Responses were scored on a 5‐point Likert scale ranging from 1 (strongly disagree) to 5 (strongly agree). In this study, the Cronbach's alpha was 0.81.

Job satisfaction was assessed using the 3‐item job satisfaction subscale of the Michigan Organizational Assessment Questionnaire developed by Cammann (1983) and later validated by Bowling and Hammond (2008). Responses were scored on a 5‐point Likert scale ranging from 1 (strongly disagree) to 5 (strongly agree), with higher scores indicating higher levels of job satisfaction. In this study, the Cronbach's alpha was 0.81.

Organizational commitment was assessed using the 5‐item Affective Commitment subscale of the Organizational Commitment Scale developed by Meyer et al. (1993) and later validated by Lee et al. (2001). Responses were scored on a 5‐point Likert scale ranging from 1 (strongly disagree) to 5 (strongly agree), with higher scores indicating higher levels of organizational commitment. In this study, the Cronbach's alpha was 0.84.

Based on previous research on workplace incivility in healthcare (Aman‐Ullah et al., 2023; Shi et al., 2018) and careful consideration of the relevance to the research aims, we also collected demographic information including age, gender, education, unit, years of nursing experience, and unit and hospital tenure.

### Ethical considerations

Participants were informed about the purpose of the study, emphasizing its voluntary nature and their right to withdraw. They were also assured of the confidentiality and anonymity of their responses, with a commitment to data protection and privacy. Also, to minimize response bias and ensure candid responses, we emphasized that their survey responses would not be shared with their employers. Participants were also informed about the rigorous data handling protocols, including secure data storage and access controls in order to safeguard the confidentiality of the survey data. In line with the ethical standards of research involving human subjects, the study strictly adhered to the guidelines set forth in the Declaration of Helsinki. The study was approved by the Yonsei University Health System Ethics Committee (4‐2023‐0645).

### Data analysis

Descriptive statistics and Pearson's bivariate correlations were conducted using SPSS Version 29.0. Confirmatory factor analysis and structural equation modeling were performed using Mplus version 7.0 to assess the measurement and hypothesized models. Model fit was evaluated using several indices: the standardized root mean square residual (SRMR < 0.08), comparative fit index (CFI > 0.90), Tucker–Lewis Index (TLI > 0.90), and root mean square error of approximation (RMSEA < 0.08) (Browne & Cudeck, 1992). When testing the structural model, we controlled for hospital tenure and unit tenure in years due to their statistically significant correlations with intention to leave (*r* = −0.22, *p* < 0.01 and *r* = −0.18, *p* < 0.01, each), job satisfaction (*r* = 0.14, *p* < 0.01 and *r* = 0.14, *p* < 0.01, respectively), and organizational commitment (*r* = 0.18, *p* < 0.01 and *r* = 0.14, *p* < 0.01, respectively). The significance of indirect paths was tested using bootstrapping with 10,000 samples and 95% bias‐corrected confidence intervals (CI) because an alternative method, the Sobel test, incorrectly assumes normality of the indirect effect (Preacher & Hayes, 2008).

## RESULTS

### Sample characteristics

The participants were predominantly female (*n* = 1,180, 94.0%) with a mean age of 31.2 years (*SD* = 6.3) and an average of 7.5 years of nursing experience (*SD* = 6.3). Their average hospital tenure was 6.7 years (*SD* = 6.0) and the mean unit tenure was 4.6 years (*SD* = 4.3). The majority of participants (*n* = 1,251, 99.7%) held permanent positions, and most (*n* = 1,169, 93.1%) earned a baccalaureate or higher degree. The largest group of nurses (*n* = 730, 58.1%) worked in medical, surgical, and combined medical‐surgical units, with the rest in specialized areas including emergency, critical care, and perioperative units. Around 65% of the nurses (*n* = 817) worked in hospitals located in metropolitan regions, and over 80% (*n* = 1,022) worked in private hospitals.

### Preliminary analyses

Pearson's bivariate correlation analyses demonstrated that both workplace incivility and workload had positive relationships with nurse burnout (Table 1). Workplace incivility and workload were negatively associated with job satisfaction and organizational commitment but positively associated with turnover intention. Burnout showed positive associations with turnover intention and negative associations with job satisfaction and organizational commitment. The variance inflation factors, between 1.07 and 1.63, showed no multicollinearity issues (Hair et al., 2010), and skewness and kurtosis levels (ranging from −0.42 to 0.27) indicated normality (Curran et al., 1996).

**TABLE 1:** Correlations and descriptive statistics for key study variables (*N* = 1,255).

Variable	1	2	3	4	5	6
1. Workplace incivility	–					
2. Workload	0.16**	–				
3. Burnout	0.26**	0.60**	–			
4. Turnover intention	0.21**	0.31**	0.44**	–		
5. Job satisfaction	−0.26**	−0.34**	−0.57**	−0.54**	–	
6. Organizational commitment	−0.33**	−0.18**	−0.32**	−0.38**	0.53**	–
*M*	2.01	3.86	3.69	3.15	3.18	3.40
*SD*	0.56	0.80	0.71	0.86	0.74	0.73

Abbreviations: *M*, mean; *SD*, standard deviation.

** *p *< 0.01.

### Measurement model

Confirmatory factor analysis was performed to test the discriminant validity of the six‐factor measurement model. All the observed variables had statistically significant loadings ranging from 0.47 to 0.91, surpassing the threshold of 0.40 (Browne & Cudeck, 1992). The measurement model also showed adequate fit to the data [χ^2^ (215) = 1,164.466, *p* < 0.001; CFI = 0.934, TLI = 0.922, SRMR = 0.043, and RMSEA = 0.059].

### Hypothesized model

**FIGURE 1:**
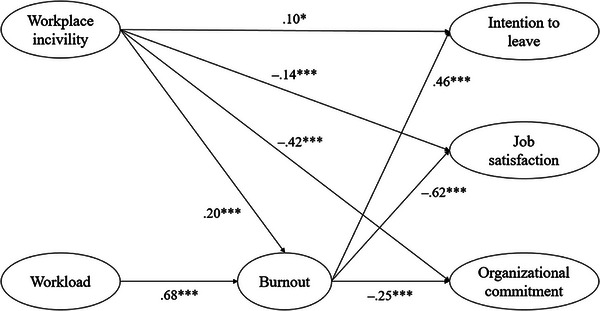
Structural equation modeling (partial) results with standardized coefficient estimates. *Note*. Insignificant paths are not displayed for parsimony. *p < 0.05, ***p < 0.001.

Our hypothesized partial mediation model showed good fit indexes [χ^2^ (255) = 1,354.328, *p* < 0.001; CFI = 0.924, TLI = 0.911, SRMR = 0.053, and RMSEA = 0.059]. As displayed in Figure 1, statistically significant path coefficients were observed among the six study variables. We tested an alternative model by excluding the six direct paths from the predictor variables to the outcome variables to specify a full mediation model. This model also provided a decent fit [χ^2^ (261) = 1,464.69, *p* < 0.001; CFI = 0.917, TLI = 0.905, RMSEA = 0.061, SRMR = 0.061]. A Chi‐square difference test was performed to compare the two models, and the result confirmed that the model fit of the partial mediation was significantly better than that of the full mediation model (∆ χ^2^ = 110.36, ∆ *df* = 6, *p* < 0.05).

As shown in Figure 1, workplace incivility and workload both had positive significant effects on burnout, but the effect of workload (β = 0.68, *p* < 0.001) was stronger than that of workplace incivility (β = 0.20, *p* < 0.001). Moreover, burnout was positively associated with turnover intention (β = 0.46, *p* < 0.001) and negatively related to job satisfaction (β = −0.62, *p* < 0.001) and organizational commitment (β = −0.25, *p* < 0.001). Also, the direct effects of workplace incivility on the three outcome variables were all statistically significant. Specifically, workplace incivility reduced job satisfaction (β = −0.14, *p* < 0.001) and organizational commitment (β = −0.42, *p* < 0.001) but increased turnover intention (β = 0.10, *p* < 0.05). Conversely, the direct effects of workload on the three work attitude variables were all nonsignificant. Using bootstrapping analysis with 10,000 samples, the study estimated the indirect effects of workplace incivility and workload on turnover intention, job satisfaction, and organizational commitment via burnout. All six tested mediation effects were significant, with 95% CIs not including zero (Table 2). Workplace incivility reduced job satisfaction and organizational commitment, and increased turnover intention, both directly and indirectly through burnout. Workload, however, only influenced these outcomes indirectly through burnout.

**TABLE 2:** Standardized coefficients of all direct and indirect effects in the hypothesized model (*N* = 1,255).

Path	β	*p*	95% CI [lower, upper]
**Direct**			
Workplace incivility → Turnover intention	0.10	0.029	[0.010, 0.189]
Workplace incivility → Job satisfaction	−0.14	0.001	[−0.226, −0.060]
Workplace incivility → Organizational commitment	−0.42	<0.001	[−0.500, −0.341]
Workload → Turnover intention	0.01	0.836	[−0.092, 0.114]
Workload → Job satisfaction	0.05	0.261	[−0.040, 0.146]
Workload → Organizational commitment	0.07	0.163	[−0.027, 0.158]
**Indirect**			
Workplace incivility → Burnout → Turnover intention	0.09	<0.001	[0.052, 0.125]
Workplace incivility → Burnout → Job satisfaction	−0.12	<0.001	[−0.165, −0.077]
Workplace incivility → Burnout → Organizational commitment	−0.05	<0.001	[−0.071, −0.027]
Workload → Burnout → Turnover intention	0.31	<0.001	[0.231, 0.383]
Workload → Burnout → Job satisfaction	−0.42	<0.001	[−0.492, −0.351]
Workload → Burnout → Organizational commitment	−0.17	<0.001	[−0.239, −0.098]

*Note*. CI, bias‐corrected confidence interval.

## DISCUSSION

This study examined the impact of workplace incivility and workload on nurses' turnover intention, job satisfaction, and organizational commitment, with the mediating role of burnout. We found that workplace incivility and workload both positively affected burnout. This result is in agreement with the previous research demonstrating that workplace incivility generates job burnout (Aman‐Ullah et al., 2023; Shi et al., 2018) and that excessive workload was positively related to burnout among nurses (Diehl et al., 2021). This current study extends the prior literature by demonstrating that, even when controlling for each other, both workplace incivility and workload significantly exacerbate burnout.

Moreover, consistent with previous research, we found that burnout significantly decreased job satisfaction (Jun et al., 2021) and organizational commitment (Wullur & Werang, 2020), but increased turnover intention (Aman‐Ullah et al, 2023). Additionally, the hypothesized mediation effects were significant, indicating that both workplace incivility and workload indirectly increased turnover intention and decreased job satisfaction and organizational commitment through increased burnout. This finding highlights the validity of the SSO framework (Koeske & Koeske, 1993) in explaining how workplace incivility and workload affect work attitudes through the mechanism of heightened job strain, specifically burnout.

Our mediation analyses revealed intriguing findings regarding the impact of workplace incivility and workload on intention to leave, job satisfaction, and organizational commitment. Surprisingly, even after considering their indirect effects, workplace incivility demonstrated a significant direct influence on these work attitude variables, whereas workload did not exhibit any significant direct effects. In other words, workplace incivility not only indirectly affected the work attitude variables by increasing burnout, but it also directly affected them independently. This finding indicates that the presence of workplace incivility alone may worsen nurses’ intention to leave and decrease job satisfaction and organizational commitment, regardless of its impact on burnout. On the other hand, workload did not demonstrate significant direct effects but influenced work attitudes through increased burnout, which contradicts previous research where workload had a direct significant effect on work attitudes (e.g., Tayfur Ekmekci et al., 2021). Our interpretation of these findings is that workload may have a more prolonged impact on nurses’ work attitudes compared to the more acute and sporadic nature of incivility, as described by Smith et al. (2018). Chronically heavy workloads may create cumulative strain on nurses through ongoing physical, cognitive, and emotional demands (Ivziku et al., 2022). Where an effective nurse leader can intercede proactively and directly to quell harmful incivility among staff, there are many structural or systemic workplace factors that contribute to workload, such as the staffing complement (e.g., numbers, skill mix, and experience), the acuity and dependency of patients, the physical layout or geography of the work environment, and the presence/or absence of resources and supports (e.g., administrative help, educators, and managers) (Ivziku et al., 2022; MacPhee et al., 2017). Given the multiple factors at different systems levels that influence workload, nurse leaders may be unable to intervene as effectively to reduce workload and burnout as incivility among staff.

Our findings underscore the necessity for healthcare organizations to address both workplace incivility and high workload to improve nurses’ work attitudes. With respect to workplace incivility, one US study (Smith et al., 2018) identified a significant inverse relationship between “healthy” work environments and incivility. This study highlighted the crucial role of effective nurse managers in maintaining professional standards and fostering positive workplace relationships. Organizational strategies to enhance workplace civility include training programs for managers and staff to recognize and prevent incivility; enforced, zero tolerance policies; and accessible procedures for handling complaints (Abdollahzadeh et al., 2017). Regarding workload, although there are numerous systemic challenges associated with different physical, cognitive, and emotional demands on nurses, recent research indicates how leaders can use concrete strategies that enhance nurses’ positive perceptions of their work environments and consequently, lessen their perceived workload demands. For example, nurses who reported having a supportive team and who received recognition from their supervisor reported less burnout from work demands than nurses who lacked these supports (Diehl et al., 2021). Nurse leaders can also monitor the types of workplace demands that create the most undue stress for nurses: documenting and addressing high‐stress demands on behalf of nurses, and making timely adjustments to resources whenever possible (Shan et al., 2022).

### Limitations

This study has several limitations. First, the data were derived from direct care Korean nurses working in general hospitals. Caution should be exercised when generalizing the findings to other healthcare populations’ contexts. Second, the cross‐sectional design employed in this study precludes establishing causality between the variables under investigation. Third, although we informed participants about the anonymity and confidentiality of responses to minimize potential biases and ensure credibility of findings, relying on self‐reported data from nurses may have introduced response bias, which might have affected the accuracy of the results.

### Implication for nursing and health policy

With the rise of workplace incivility and discriminatory behaviors toward others, organizations and their leadership need to promote values and professional standards of care (e.g., equity, diversity, and inclusivity), which are associated with an ethical, organizational identity (Sekerka & Yacobian, 2019). There are educational approaches (e.g., case‐based learning with ethical decision‐making models) and leader role‐modeling that can raise employees’ respect and appreciation for diverse others (Phillips et al., 2018). Organizational metrics and regular assessments are needed to provide data‐driven evidence of workplace incivility (Atashzadeh‐Shoorideh et al., 2021; Sekerka & Yacobian, 2019), while nursing shared governance councils and workplace civility committees are needed to empower nurses and to promote their active roles in addressing stressful, uncivil behaviors (Yoder‐Wise, 2018).

Another workplace stressor, heavy workloads, creates chronic strain that eventually manifests as burnout and turnover. A major reason for heavy workloads is poor “fit” between patient care needs and nurse staffing (MacPhee et al., 2017). Recent research has shown how nurse‐driven assessments of patients’ acuity and dependency needs can provide accurate, objective data for determining nurse staffing complements, promoting more manageable workloads (MacPhee et al., 2017). Greater nurse control over patient care delivery and staffing determinations may lessen burnout (Havaei et al., 2022).

## CONCLUSIONS

Our findings highlight the distinct contributions of workplace incivility and workload in explaining variations in nurses’ turnover intention, job satisfaction, and organizational commitment. This study makes a valuable contribution to the existing literature by shedding light on the distinct mechanisms underlying the effects of the two types of workplace stressors on various work attitudes via burnout. Our study findings also indicate that chronic workload stressors may have a greater impact on nurse burnout than acute, sporadic episodes of workload incivility. By exploring the mediation model, the study fills research gaps on how incivility and workload jointly affect burnout and nurses’ work attitudes.

## AUTHOR CONTRIBUTION

*Study design*: SEL, JS. *Data collection*: SEL. *Data analysis*: JS. *Study supervision*: SEL. *Manuscript writing*: JS, SEL, MM. *Critical revision for important intellectual content*: SEL, MM.

## CONFLICT OF INTEREST STATEMENT

The authors declare no conflicts of interest.
